# The American Academy of Dental Surgery. Proceedings of Special Meetings, October 19 and 20, 1875

**Published:** 1876-01

**Authors:** 


AMERICAN JOURNAL

OF

DENTAL SCIENCE.

Vol. IX. THIRD SERIES—JANUARY, 1876. No. 9.

### ARTICLE I.

### *The American Academy of Dental Surgery.*

Special meetings of October 19th and 20th, 1875.

*Reported for the American Journal of Dental Science.*

*Evening Session.*—Continued.

On the evening of the 19th, the Academy assembled at the house of the President, No. 50 West 35th Street. The meeting was called to order at about 7-30 P. M., and the regular business proceeded with. A number of dentists were elected either active or corresponding members. Presentations were made to the library, by Dr. Samuel Welchens, Lancaster, Pa ; Dr. N. M. Beckwith, New York ; Dr. Geo. H. Perine ; and to the Museum by Dr. C. H. Eccleston, Oxford, New York ; Dr. F. S. Howard, New York ; Dr. John S. Smith, Columbia, Pa. Additional letters of a complimentary character were received from a large number of prominent dentists. Papers were received from the following gentlemen, but the programme being full, they were reserved for a reading before a future meeting of the Academy: Dr. E. L. Hunter, Enfield, N. J., paper upon "Mechanical Abrasion of the Anterior Teeth," Dr. Geo. A. Mills, Hartford, Ct., paper upon "Association."

The essay of the evening was then read by the author, D. D. Miles, M. D., Phila. Pa.; it was entitled, "Preservation of the Teeth as a Specialist."

Dr. Miles said that all the attention heretofore given to this subject had been an incidental feature of dentistry; but if it was a field large enough and sufficiently important to occupy the whole attention of any one practitioner, under the *ægis* of due qualifications, why should it not be occupied as such? Division of labor had been elsewhere found favorable to high special attainments; experts were found devoting their whole time and attention to their particular specialties; and even the barber had employees who were considered adepts in some particular feature of the business. A very fine plugger or operator ought never to touch a plate, as it would spoil his hands for that acute sense of touch which was needed in the delicate manipulations necessary in operating upon sensitive cavities. A hobby in any one avocation is the most constant incentive to improvement, and a desire to excel in any particular direction, results in profitable improvement.

The protection and preservation of good teeth, together with the restoration of imperfect teeth, should be the legitimate field of the specialist; and his attention and supervision should be always in demand. The fact is, that the most apparent efforts for the preservation of the teeth have been monopolized by enterprising venders of empirical compounds.

Absolute purity should be the basis upon which treatment must be founded. To establish this necessary foundation, the cleansing process should be effected by means differing as much as the different phases of discoloration—accumulation of tartar—different conditions of the gums—from simple irritation and softening to phogedemic ulcers. These require various topical treatment; some require powerful action, as the dilute re-agents, escharotic stiptics —down to antiseptic astringents, all acting as tonics and alteratives,—down to the most gentle anodynes, and fragrant stimulants. In the subsequent treatment, and for the use of the patient, different materials of erasive and determinative qualities should be discriminatingly used, down to an inpalpable polish and neutralizer, to answer the different conditions and constitutional habits, whether of an acid or alkaline re-action,—all cleansing, polishing, hardening and strengthening the parts, the bony as well as the soft tissues. Treated so, and in time, the teeth would be pure, hard and beautiful, the gums dense and strong. No more impurity, no more bad teeth.

No preparation could be *sold over a counter* which would accomplish this. The general dentist could not, as a rule, spend the time necessary to do it. The field should be occupied by the specialist, as only through him could the best results be attained.

The discussion of this paper was postponed until later in the evening, in order to witness an exhibition of cauterization by the galvanic battery. The use of the battery as a cautery was shown by applying the platinum wire to the leg of a rabbit, (whose spinal cord was first punctured,) when the wire passed through flesh and bone, and made a complete amputation without hæmorrhage.

Dr. Geo. H. Perine, in referring to the battery, said that the dentists had endeavored to steal the thunder of the doctors in certain operations, and especially in operations upon the oral cavity, had they met with success. He had made several very satisfactory operations. They might perform the operations free from shock, safe from hæmorrhage. They might employ the cautery for many purposes —for the destruction of nerves,—for the taking away of diseased tissue—even for the removal of bone. The instrument should be used with great care, and be brought to a white heat when removing sensitive dentine. The operator should be careful not to attach the instrument to a tooth not to be operated upon, and not to let it touch any part of the tooth except that to which he wanted to apply the heat.

Prof. Wm. A. Hammond, said that the application of electricity to surgery was not exactly in his line ; but some ten years ago he had used the cautery in a little difficult case with success. The patient had a fistulous opening in the palate. Those who had tried to close a fistulous opening in the palate, knew how much more apt they were to wound the soft parts that were healthy than to get the needle into the hole. After the loop had been introduced into the opening, however, the circuit was closed by a little mechanism, and the operation completed. While he was not now so enthusiastic in his ideas of the medical value of the battery as he used to be, yet he still thought that the applications of it to surgery would become more extensive, especially in operations about the mouth. For the removal of certain kinds of aneurisms, it was certainly of great value.

At the conclusion of these remarks, the paper of Dr. E. D. Miles was discussed.

Dr. A. P. Merrill said that the preservation of the teeth was a part of dentistry which was somewhat neglected. The labor was poorly paid for. By an effort on the part of the profession, the scale of prices for cleaning the natural teeth could be generally advanced, and made to pay them to attend to the matter.

Dr. Perry said that he had established in his practice the rule to charge by the hour in all operations, and in that way the cleaning of teeth was just as profitable to him as filling them.

Dr. J. M. Riggs said he feared the dentists were running into specialties too much. If the thing continued, patients would soon be running from one street to another, first to one specialist, and then to another. Very few dentists, however, spent the proper time in cleansing the teeth. Brown tooth powder should not be used ; it would break down the coat of enamel. The cleansing of the teeth was neglected, because the dentist did not get enough for the work. The question was often asked: "What do you charge for cleaning teeth?" It was a common answer: "Fifty cents!" [Laughter.] They could go into half a dozen offices in Hartford, Ct., and get their teeth cleaned for fifty cents. No operator wanted to spend his time in polishing the teeth, and stopping the secretion that lodged upon the gums, without a fair remuneration for the work.

In cleaning these teeth, he shaped a stick, about the size of a pipe stem into a wedge—shaped chisel about one eighth of an inch wide, and dipping the stick into a mixture of pulverized pummice stone and water, polished the surface of the tooth, until he had removed all the roughness about the gum; and when there was a disposition for the brown coating to reappear upon the tooth, he would go over it a second time,—a third time being rarely needed. This could not be done in fifteen minutes, nor for fifty cents. [Laughter.]

The teeth should be attended to in time. If they were all jumbled up together—very much crowded—some popping out above the arch of the jaw, the laterals perhaps depressed, or half of the six year molars in or broken down, by extracting these at the proper time, the bicuspids would fall back, and the front of the mouth would be brought into the most regular and beautiful order.

Adjourned to meet at the College of Physicians and Surgeons, at ten o'clock on the following morning.

---

### Second Day.—*Morning Session.*

The Academy assembled in the lecture-room of the College of Physicians and Surgeons, at 10 o'clock on the morning of the 20th, to proceed with the second day's work. The President called the meeting to order, and the Rev. Mr. Pratt, of New York, opened the proceedings with prayer. A number of letters had been received by the morning mail, and some of them were read. The first paper read was upon the subject of "The Dental Pulp; its Pathology and Treatment, by W. B. Willmott, D. D. S., L. D. S., F. A. A. D. S., Toronto, Canada.

Rev. Mr. Pratt read the paper for the author, who was unable to be present on account of sickness. We give the paper in full. (See article on "The Dental Pulp," in this number of JOURNAL.)

*Discussion.*

Dr. Aba. Robinson, Georgetown, Mass., said that Dr. Willmott's paper was the best upon the subject that he had ever heard. He had felt a deep interest in it, for the reason that he was one of the early ones to advocate the preservation of the pulp where it was exposed. He advocated the matter before a dental society as far back as 1851. A set of aphorisms had been introduced by members of the society at a meeting in that year. The chairman of the committee on those aphorisms was Dr. E. J. Dunning. It was said that when a pulp was exposed, the tooth was lost. He would not consent that those aphorisms should be passed without opposition. At that time he had practiced the preservation of the teeth when the pulp had been recently exposed. He adopted a method of treatment that was almost uniformly successful. He first bored the tooth, then dropped upon it two drops of collodion, which left a smooth elastic coating over the exposed part; then he would take a few folds of foil, and lay it upon the part, and around it, avoiding pressure, and so leave it without other protection, until he could get far enough above it to leave a solid filling. In nine cases out of ten, he saved those teeth without further trouble.

Dr. Samuel Welchens, Lancaster, Pa., said he took pleasure in adding his testimony to the excellence of the paper just read. There was nothing for him to discuss in the paper except where the writer spoke of using the chloride with creosote. He had often found occasion to use creosote, but he used the oxy-chloride of zinc with the creosote. After devitalizing the pulp, where it sloughed, he invariably extirpated the whole of it, and filled the foramen either with oxy-chloride of zinc, or Guilloi's cement. He would then cover with metallic filling.

Dr. W. S. Elliott, Goshen, N. Y., said that in the treatment of the dental pulp practitioners differed. Some used oxy-chloride of zinc; others used substances such as sponge, wood, paper, etc., but in nine cases out of ten they would say that they used creosote. Why did they use creosote? What was its prophylactic action upon the pulp? That was the point upon which he wanted to give his theory. Creosote precipitated albumen. The pulp being held in contractive tissue, the first phenomenon upon the application of creosote was contraction, and as the blood column returned and filled the vessels, it came in contact with the creasote, and the effect was the precipitation of the albumen. In the precipitated albumen was a mass of entangled fibrin. This was nature's first step in the construction of new tissue. The fibrin having vital properties of its own, was able to carry on the function of nutrition to a certain extent; to this was united the function of the red corpuscles; and they worked upon the precipitated albumen, which was a floor for the new tissue to be built upon. If the parts had not been too much disturbed in the first place, nature would take up the organized soft tissue, the lime salts would be deposited, and secondary dentine would be formed. That was the theory.

The next paper read was entitled: "Necessity of Dentists in the Army and Navy." By A. P. Merrill, D. D. S., F. A. A. D. S., New York.

He said that the subject of his paper had been under consideration by both Houses of Congress since "1868," when the first bill was introduced by Senator Hamlin, of Maine. Although no action was taken, other than having it referred to the committee on military and naval affairs, yet the importance of some legal provision to meet this much needed requirement had been so agitated that partial success was already obtained. That skilled dental surgeons had been essential at West Point and Annapolis all agreed and that the soldiers and sailors in the more humble sphere of the service should be equally considered had been made evident. A petition was introduced into the 42d Congress, calling attention to this matter; and upon its presentation a bill was introduced into Congress by the Hon. Dwight Townsend, and read twice, which was referred to the military committee, and urged to be reported back to the House to be taken up and passed; but as this was a new feature in the service, and selfish influences were brought to bear against it, the bill was held back.

It was time that this subject should be again taken up by the better part of our physicians and specialists, and urged forward. In France the subject had been considered favorably, and it had also been brought before the government of England, and sanctioned by her best military officers and surgeons. No government could afford in this enlightened nineteenth century, while demanding of its soldiers good sound teeth on entering its service, to place them where they could not have the advantage of first-class dental skill.

*Discussion.*

Dr. Geo. H. Perine, said that some of those present would remember that during his editorial connection with with a dental journal, in 1858, he had called the attention of the profession to this subject. He thought that the French originated the question, but the American dentists were the first to give it character. The English and French governments had partially recognized the necessity of dentists in the army and navy. It was not the officers of the army and navy that required the services of army dentists so much as the common soldiers. The officers were able to pay for the dentists' services, but the soldiers were not. The American Academy of Dental Science had this matter in hand, and they would succeed in it. [Applause.]

Dr. I. J. Ketchan, New York, said that the matter of attending to the soldiers teeth could not be expected to be done by the surgeon, any more than any general practitioner in New York could be expected to treat a case of disease of the eye or ear. The physician could not enter the domain of the dentist without having had the special training required. The health of the soldier should be considered in every way. The Government, in surrounding a man as it did with its laws and rules, was bound to attend to his physical needs. The question was a very important one, and should be acted upon.

Dr. A. P. Merrill said that in the army the medical profession were not as liberal in regard to this question as were those outside of it. The petition he had read had been prepared through the instrumentality of some of his friends, and it had been signed by such prominent names as had been connected with the army as surgeons. By carrying this matter through, they would render a great boon to the soldiers. They had partially conquered already, and they must succeed. [Applause.]

The next paper read was entitled, "Mechanical Denistry." By C. S. Hurlbut, D. D. S., Springfield, Mass.

He said that "Mechanical Dentistry," deserved their respect, at least, if for no other reason than that it was the parent of the dental art. While the profession had made rapid strides in development and attainment, this progress had been mostly confined to operative dentistry. Mechanical dentistry had been neglected, but he thought the time was fast approaching when it would take its proper place in the estimation of the profession.

After the reading of this paper, an adjournment was had until 2 P. M., the meeting first passing a resolution tendering its thanks to the Trustees and Faculty of the College for their courtesy and kindness to the Academy, and for the encouragement they had given those who were trying to bring about the recognition of dentistry as a specialty in medicine.

*Afternoon Session.*

The proceedings were commenced by a discussion of the last paper read at the morning session, "Mechanical Dentistry," which was participated in by Dr. S. S. White, Phila.; Dr. W. S. Elliott, Goshen, N. Y.; Dr. Samuel R. Stuart, Akron, Ohio; Dr. A. W. Kingsley, Elizabeth, N. J. During the discussion Mr. Geo. J. Pack, gave some valuable and interesting information on GOLD, treating of its condition under many different circumstances, and answering many questions of the gentlemen present.

Dr. John M. Riggs, Hartford, Ct., then read a paper entitled, Suppurative Inflammation of the Gums, and Absorption of that Tissue and Alveolar Process." This was followed by a paper, by Dr. W. S. Elliott, Goshen, N. Y., upon "Physics and Physiology." The discussion of these papers was omitted for want of time.

The hour of adjournment having arrived, Dr. Geo. H. Perine, the President, said:

Gentlemen: In view of the remarks I made to you in the opening session, when I bid you heartily welcome, it is now fit, as we are about to part, that I should congratulate you upon the work you have accomplished, and upon the position which the dental profession has attained here in New York. We are just on the eve of a new era. Here in New York the medical profession are ready to receive us. Have we, as a young body, been too forward? Gentlemen, we have reason to feel that the influence of the Academy has been felt in all quarters of the globe. I am happy to be privileged to-day to say these few words to you, and I hope that before long we may again gather together, and have as equally profitable a session as this has been. Thanking you all, the Academy will now adjourn to the next regular meeting, on the third Tuesday of the coming month.

